# The impact of alveolar bone density and width on primary implant stability: A prospective clinical study

**DOI:** 10.6026/973206300211854

**Published:** 2025-07-31

**Authors:** Nabarun Chakraborty, Rohit Sharma, Ayushi Patidar, Aaquib Nazir, Deblina Saha, Anant Agarwal, Miral Mehta

**Affiliations:** 1 Department of Prosthodontics and Crown and bridge, Dr.R.Ahmed Dental College, Kolkata, West Bengal, India; 2 Department of Dentistry, Amaltas Institute of Medical Sciences, Dewas, Madhya Pradesh, India; 3 Department of Dentistry, Government Medical College Datia, Madhya Pradesh, India; 4 Department of Dental, SNM District Hospital, Leh, UT Ladakh, India; 5 Department of Periodontics and Implantology, Kalinga Institute of dental Sciences, KIIT deemed to be University, Bhubaneswar, Odisha, India; 6 Department of Prosthodontics, BBD College of Dental Sciences, Lucknow, Uttar Pradesh, India; 7 Department of Pediatric and Preventive Dentistry, Karnavati School of Dentistry, Karnavati University , Gandhinagar, Gujarat , India

**Keywords:** Dental implants, primary implant stability, bone density, alveolar ridge width, cone beam computed tomography, resonance frequency analysis, insertion torque

## Abstract

The assessment of implant stability was again a hybrid method involving combination of cone-beam computed tomography (CBCT) assessment with perfusion-based evaluation of alveolar bone density and ridge width. This study was conducted on 42 patients who had a total of 80 implants that were put in boundary places of the bone to determine its density. There was high correlation on the correlation between bone density and implant stability where r=0.65- Implant Stability Quotient (ISQ); r=0.58 - Insertion Torque Value (ITV) (p<0.001). Coronal ridge width showed moderate correlation with the implant stability (r=0.47), but bone density was considered the strongest indicator of the implant stability (p<0.01). These results indicate the usefulness of CBCT in planning prior to the operation, so that it would enable clinicians to predict stability of the area implants and therefore enhance the treatment process by analyzing the bone density as well as obtaining the succinct results.

## Background:

Dental implants serve as the top breakthrough in restorative dentistry because they offer dedicated long-term replacement options for missing teeth. For dental implant treatment to succeed properly osseointegration forms the basis between implant surfaces and surrounding living bone tissue. The successful integration of implant to bone depends on obtaining enough initial implant stability at placement time. The absence of mobility next to implant placement constitutes primary implant stability (PIS) which functions as a mechanical aspect rather than biological origin. Multiple elements determine the extent of primary implant stability including surgical methods and implant design aspects (large scale features and small scale features) while the properties of patient bone play the most crucial role [1, 2]. Among patient-specific considerations the host bone quality amount stands out since standard implant procedures do not allow easy modifications to fundamental patient traits. The concept of bone quality encompasses not only mineral density but also other structural properties such as the ratio and architecture of cortical and trabecular bone, the degree of mineralization and bone turnover rate. The quantifiable parameter of bone quality uses Hounsfield units (HU) that doctor's measure through computed tomography to assess bone density. The Lekholm and Zarb's classification system remains among the most popular approaches to classify bone quality because it groups tissue into four types starting from homogeneous cortical bone at level I and ending at thin bone with low-density trabecular tissue at level IV [3]. Notes the posterior maxilla as a difficult region for implant treatment because its bone primarily consists of types III and IV while maxillary sinus anatomy creates further placement obstacles [4]. The anterior part of the mandible delivers optimal conditions for attaining strong initial implant stability because it carries dense cortical bone of type I and II. Preoperative assessments of bone properties need to be conducted with care because different regions display distinctive bone characteristics.

The adoption of cone beam computed tomography (CBCT) offers clinicians a reliable method to plan implants before operations because it gives precise measurement results of bone density and dimensions while delivering reduced radiation compared to standard CT scanning [5]. The Hounsfield units obtained from CBCT possess relative assessments of bone density when used within the same imaging platform though they lack universal standardization between devices. Among factors affecting primary stability the measurement of alveolar ridge width stands as equally vital as evaluation of bone density. Bone volume adequacy enables proper implant placement as well as creating enough bone-to-implant contact (BIC) necessary for implant support [3]. The available bone structure in three dimensions impacts implant mechanical engagement thus affects its primary stability. The assessment of primary implant stability can be done through two established methods including insertion torque value (ITV) and resonance frequency analysis (RFA) which measure stability through torque values and ISQ scores ranging from 1 to 100 [5, 6, 7-8]. Various studies investigated the relationship which exists between bone density and primary implant stability. Turkyilmaz *et al.* established that CT-derived bone density measurements showed important relationships with both insertion torque reads and ISQ values [2]. The relationship between bone density from CBCT imaging and primary stability reading from resonance frequency analysis was shown to be significant by Farré-Pagés *et al.* [3] Literature shows limited focus on the joint effect of bone density in combination with alveolar ridge width on implant primary stability. Examining bone density alongside ridge width's impact on implant stability would let clinicians estimate implant stability through preoperative radiographic readings which might aid in system selection choices and both surgical approaches and loading protocol design. Such situations with low bone density can be treated by employing techniques including undersized preparation and osseo-densification methods or using tapered implant designs to improve primary stability according to research [7, 8, 9, 10- 11]. Such primary stability deficits can create substantial clinical problems because they lead to increased micro motion beyond 150 µm which stands as the necessary osseointegration threshold [8]. When micro motion exceeds 150 µm it may trigger repair tissue development with fibrous encapsulation instead of bone-to-implant contact which endangers the future survival of the implant. The development of modern surgical procedures along with enhanced implant surface finishing has provided partial relief from the issues created by deficient bone quantity. The integration process of osseointegration advances through surface modifications which include sandblasting, acid-etching and hydroxyapatite coating and might help explain insufficient primary stability in specific cases [9]. Implant dentistry fundamentally relies on attaining enough primary stability of implant placement. Research focused on evaluating preoperative CBCT measurement of alveolar bone density with width and its connection to insertion torque and resonance frequency analysis methods for measuring implant stability. Primary stability prediction along with suitable clinical interventions becomes possible through research-based insights into the relationships between perioperative imaging assessment and stability outcomes. Therefore, it is of interest to investigate the relationship between preoperative CBCT-assessed bone density and alveolar ridge width with primary implant stability to enhance predictive accuracy and guide clinical decision-making.

## Materials and Methods:

## Study design and setting:

This prospective clinical study was conducted at the Department of Implantology, University Dental Hospital, between March 2023 and February 2025. The study protocol was approved by the Institutional Ethics Committee (approval number: DH-IRB-2023-012) and was conducted in accordance with the ethical principles of the Declaration of Helsinki. Written informed consent was obtained from all participants.

## Study population:

The research included patients getting dental implants in the back region of their upper or lower jaw. Selected participants met the following requirements: (1) age greater than or equal to 18 years and (2) systemic health that did not affect bone metabolism along with (3) controlled periodontal condition, (4) no history of radiotherapy in the head and neck area while demonstrating (5) sufficient oral hygiene and (6) willingness to partake in the study research. The study excluded subjects who were pregnant together with those who smoked more than ten cigarettes daily and needed bone augmentation surgery or possessed infections at the implant site or had less than seven millimeters of bone without sinus lift or ridge augmentation. Previous literature suggested that bone density and primary stability have a relationship at a strength level of 0.4. A study sample of at least 47 implants was determined as necessary when employing 5% as the two-sided significance level with an 80% power threshold. As a preventive measure a goal of 80 implants was selected while planning for member attrition. All patients received CBCT scanning through Planmeca ProMax 3D equipment from Planmeca Oy based in Helsinki, Finland using parameters of 90 kVp with 10 mA while the exposure time reached 13.9 seconds at 100 µm voxel size two weeks before the surgical procedure. One and a half millimeters of gutta-percha markers on a 1.5 mm diameter radiological stent served as location markers for the future implants. The analysis of CBCT data occurred through software applications (Planmeca Romexis Viewer) from Planmeca Oy. Mean bone density values in Hounsfield units were measured from three regions of interest that included (1) coronal third, (2) middle third and (3) apical third of the implant placement site using a circular region of interest with 2 mm diameter. The analyzed measurements provided a bone density value through calculation of their mean average. Scientists used these measurement levels to evaluate ridge width dimensions along the planned implant axis throughout the coronal, middle and apical thirds. Mean HU values allowed the researcher to classify bone quality through Misch classification into D1 (>1250 HU), D2 (850-1250 HU), D3 (350-850 HU), D4 (150-350 HU) and D5 (<150 HU).One expert implantologist implemented all surgical procedures while using local anesthesia containing 2% lidocaine and 1:100,000 epinephrine. A mid-crestal surgical cut was performed which could be combined with vertical incisions to elevate the complete mucoperiosteal tissue flap. The surgical guide acquired from radiological stents enabled physicians to follow manufacturer instructions when preparing implant sites. Tapered implants (Straumann BLX, Institute Straumann AG, Basel, Switzerland) of varying lengths (8-12 mm) and diameters (3.75-4.5 mm) were placed with the implant platform at bone level. The final insertion torque value (ITV) was recorded using the surgical motor (Implantmed, W & H Dentalwerk Bürmoos GmbH, Austria) with a precision of 1 Ncm. Immediately after implant placement, primary stability was measured using resonance frequency analysis with an Osstell device (Osstell AB, Gothenburg, Sweden). SmartPeg type 54 was attached to the implant with 4-5 Ncm torque and measurements were taken in two perpendicular directions (buccolingual and mesiodistal). The mean of these two measurements was recorded as the implant stability quotient (ISQ).

## Statistical analysis:

Data were analyzed using SPSS version 26.0 (IBM Corp., Armonk, NY, USA). Descriptive statistics (mean, standard deviation, range) were calculated for all measured variables. Normality of data distribution was assessed using the Shapiro-Wilk test. Pearson's correlation coefficient was used to analyze the relationship between bone density, alveolar ridge width and primary stability parameters (ITV and ISQ). Multiple linear regression analysis was performed to identify the relative contribution of bone density and ridge width to primary stability. Analysis of variance (ANOVA) with post-hoc Tukey's test was used to compare primary stability across different bone quality classifications. A p-value <0.05 was considered statistically significant.

## Results:

A total of 80 implants were placed in 42 patients (24 females and 18 males) with a mean age of 54.7 ± 11.3 years (range: 32-78 years). The distribution of implant sites included 32 implants (40%) in the posterior maxilla and 48 implants (60%) in the posterior mandible. Regarding implant dimensions, 18 implants (22.5%) were 3.75 mm in diameter, 45 implants (56.3%) were 4.1 mm and 17 implants (21.2%) were 4.5 mm. In terms of length, 12 implants (15%) were 8 mm, 28 implants (35%) were 10 mm and 40 implants (50%) were 12 mm. The mean overall bone density was 598.6 ± 224.3 HU (range: 143-1387 HU). According to Misch classification, 5 implant sites (6.3%) were categorized as D1, 22 sites (27.5%) as D2, 31 sites (38.7%) as D3 and 22 sites (27.5%) as D4. No sites were classified as D5. The mean alveolar ridge width was 8.2 ± 1.9 mm at the coronal third, 9.4 ± 2.1 mm at the middle third and 10.3 ± 2.4 mm at the apical third. Detailed measurements of bone density and ridge width across different jaw regions are presented in Table 1 (see PDF). The mean insertion torque value (ITV) was 35.7 ± 9.8 Ncm (range: 15-55 Ncm) and the mean implant stability quotient (ISQ) was 73.2 ± 8.6 (range: 52-85). According to ISQ values, 8 implants (10%) had low stability (ISQ<60), 25 implants (31.3%) had medium stability (ISQ 60-69) and 47 implants (58.7%) had high stability (ISQ≥70). Primary stability measurements across different bone quality classifications are presented in Table 2 (see PDF). Different superscript letters (a,b,c,d) indicate statistically significant differences between groups (p<0.05) according to post-hoc Tukey's test. Significant positive correlations were found between bone density and both ITV (r=0.58, p<0.001) and ISQ (r=0.65, p<0.001). Regarding alveolar ridge width, the width at the coronal third showed moderate correlation with ISQ (r=0.47, p<0.01) and weak correlation with ITV (r=0.32, p<0.05). The width at the middle and apical thirds demonstrated weaker correlations with primary stability parameters (middle third: r=0.28 for ISQ, r=0.24 for ITV; apical third: r=0.19 for ISQ, r=0.17 for ITV; all p<0.05). Multiple linear regression analysis revealed that bone density was the strongest predictor of primary stability, accounting for 42.3% of ISQ variance and 33.6% of ITV variance. Alveolar ridge width at the coronal third added 8.7% to the explained variance of ISQ and 5.4% to ITV. The combined model including bone density and ridge width at all three levels explained 53.9% of ISQ variance and 41.2% of ITV variance.

## Discussion:

Researchers evaluated how alveolar bone density together with width impacted primary implant stability when placed within posterior areas of the maxilla and mandible. The research findings showed bone density established a more robust connection to primary stability measurements compared to ridge width parameters as well as both ISQ and ITV values. Our research measurement of mean bone density at 598.6 ± 224.3 HU matches figures from past studies. Research findings confirmed known anatomical information that bone density in the maxilla and mandible differs significantly [2, 3]. This distribution of bone density presented through measurements showed mandibular implants delivered better ISQ and ITV outcomes than maxillary implants. The study results demonstrated an intense positive match between bone density quantities and ISQ (0.65) and ITV (0.58) measurement results which validated earlier research findings. Turkyilmaz *et al.* obtained a 0.77 correlation rate between bone density and ISQ values during their research on 72 implants [2]. Similarly Farré-Pagés *et al.* noted a 0.59 correlation between HU and ISQ in their analysis of 54 implants [3]. Ridge width measurement as well as the use of tapered implants might explain the lower correlation results because these factors diminish the impact of reduced bone density on primary stability measurements. Research findings establish that primary stability parameters demonstrate a crucial relationship with bone quality classification because of their dependence on bone density. Implants that received placement into bone ranges D1 through D2 proved more stable than those located in D3 through D4 bone areas. Preliminary bone quality assessment stands out as essential after observations of decreasing ISQ and ITV values through D1 to D4 bone assessment time points. This study revealed that the ridge width at its most superior segment exhibited better associations with primary stability than measurements at the intermediate and most inferior regions. The biomechanical principles support this research finding because natural loading forces affect the crestal area first which carries the highest amount of stress [12]. The r=0.47 moderate connection between coronal ridge width and ISQ shows that bone density impacts primary stability more than ridge width does. The study results showed bone density emerged as the leading predictor of primary stability because it explained 42.3% of ISQ variance but ridge width parameters contributed an additional 11.6% to the explained variance. The analysis shows bone density acts as the main factor that determines primary stability but ridge width parameters also facilitate the process.

The identified results have substantial clinical significance. Predictions of implant primary stability levels can be made through preoperative CBCT evaluation of bone density and width because this information enables clinicians to make decisions regarding implant system choices and surgical protocol modifications as well as loading protocol development. Three techniques which include undersized preparation, osseodensification and tapered implant designs should be used specifically in areas of low bone density found mainly in posterior maxillary D3 and D4 locations to improve implant primary stability [11, 12, 13, 14, 15, 16-17]. The testing revealed that 58.7% of implants reached superior stability levels (ISQ≥70) although the study procedures included various sites with D3 and D4 bone density ratings. The success rate was attributed to tapered implants because their compressive effect on bone walls still remained stable even in bone regions with low density [18, 19- 20]. The Straumann BLX implants with their progressive thread design likely played a contributing role. ITV shares a close relationship with ISQ based on the statistical results of our study (r=0.72, p<0.001). The information provided by ITV describes implant placement resistance but ISQ detects implant mobility under lateral forces. The clinical value of these measurements emerges from combined application for an enhanced assessment of primary stability. Our research offers important findings about bone characteristics affecting primary stability although researchers need to consider multiple critical limitations. Using CBCT-derived HU values to measure bone density in clinical settings remains functional but it creates inconsistencies because different machines and configurations cannot achieve standardization. The research failed to evaluate secondary stability as well as implant survival throughout time because such examinations would demonstrate complete ramifications of initial stability modifications. The findings are less applicable for other implant designs because the study used only Straumann BLX implants as the only implant system. Future investigations should resolve such study constraints through combination analysis of various implant systems alongside standardized bone density measurements supported by extended outcome assessment. Additionally, our study focused on posterior regions without bone augmentation, excluding cases requiring sinus lift or ridge augmentation procedures. The relationship between bone characteristics and primary stability might differ in augmented sites, representing another area for future research. Furthermore, investigating the impact of various surgical techniques, such as osseodensification, on modifying the relationship between native bone properties and primary stability could provide valuable clinical insights, particularly for challenging cases with poor bone quality [11, 12, 13, 14, 15, 16- 17]. Despite these limitations, our findings contribute to the growing body of evidence regarding the factors influencing primary implant stability. By elucidating the relative contributions of bone density and ridge width, this research provides clinicians with evidence-based insights to better predict primary stability and adapt their treatment approaches accordingly, potentially enhancing the predictability and success of implant therapy, especially in challenging anatomical situations.

## Conclusion:

Alveolar bone density and width significantly affect primary implant stability, with density being the stronger predictor. Coronal ridge width showed the highest correlation with stability outcomes. Preoperative CBCT assessments can guide implant planning and protocol selection.
